# Supplementary material for: Smart dynamic hybrid membranes with self-cleaning capability
Source: Nat Commun. 2023 Sep 16;14:5751. doi: 10.1038/s41467-023-41446-9 (PMC10505219; doi:10.1038/s41467-023-41446-9)
Supplement: Supplementary file 1 — Supplementary Information File [file 41467_2023_41446_MOESM1_ESM.pdf]

Electronic Supplementary Information

Smart Dynamic Hybrid Membranes with Self-Cleaning Capability

Pantuso et al.

Supplementary Methods

Gas permeation properties evaluation: experimental apparatus and procedure

The gas permeation properties of the membranes were measured by feeding single gas (CO_2, N_2, H_2) in dry conditions at different temperatures (25, 35, 40, 50 °C) and operating under trans-membrane pressure differences (2–8 bar). The core of the permeation system was a flat sheet membrane housed in a stainless-steel module sealed by means of silicon gaskets (Supplementary Figure 1). The stainless-steel module was planar with feed as input stream and permeated as the stream was leaving the module. The permeation flow rate of each gas was measured by means of a bubble-soap flow meter as a function of the feed/up-stream pressure as imposed by a forward pressure controller.

The single gas permeability was measured as the ratio between the permeating flux and the corresponding trans-membrane partial pressure difference for the ith species permeating the membrane, multiplied by the membrane thickness (Supplementary Equation 1). The ideal selectivity was calculated as the ratio of the permeances of two single gases measured at the same temperature (Supplementary Equation 2).

$$\text{Permeability} = \frac{\text{Permeating flux}_i \times \delta}{\Delta P_i^{\text{TM}}}, \text{[barrier]} \qquad (1)$$

$$\text{Ideal selectivity}_{\text{ij}} = \frac{\text{Permeance}_i}{\text{Permeance}_j} \qquad (2)$$

The flux in Knudsen diffusion can be described by the Supplementary Equation 3:

$$J_i = \frac{\pi n r^2 D_i^k \Delta P}{RT\tau\Delta z} \qquad (3)$$

J - flux through the membrane
n - number of pores in the membrane per unit of surface area
ΔP - pressure difference across the membrane
Δz - thickness of the membrane
τ - tortuosity
D^k - Knudsen diffusion.

The Knudsen diffusion is given by Supplementary Equation 4:

$$D_i^k = 0.66\, r \sqrt{\frac{8RT}{\pi M_i}} \qquad (4)$$

It is inversely proportional to the square root of the molecular weight of the gas molecule considered. Thus, the separation of two gases based on Knudsen diffusion is proportional to the ratio of the square root of the molecular weights (Supplementary Equation 5):

$$\alpha_{j,k} \propto \sqrt{\frac{M_k}{M_j}} \qquad (5)$$

Supplementary Figures

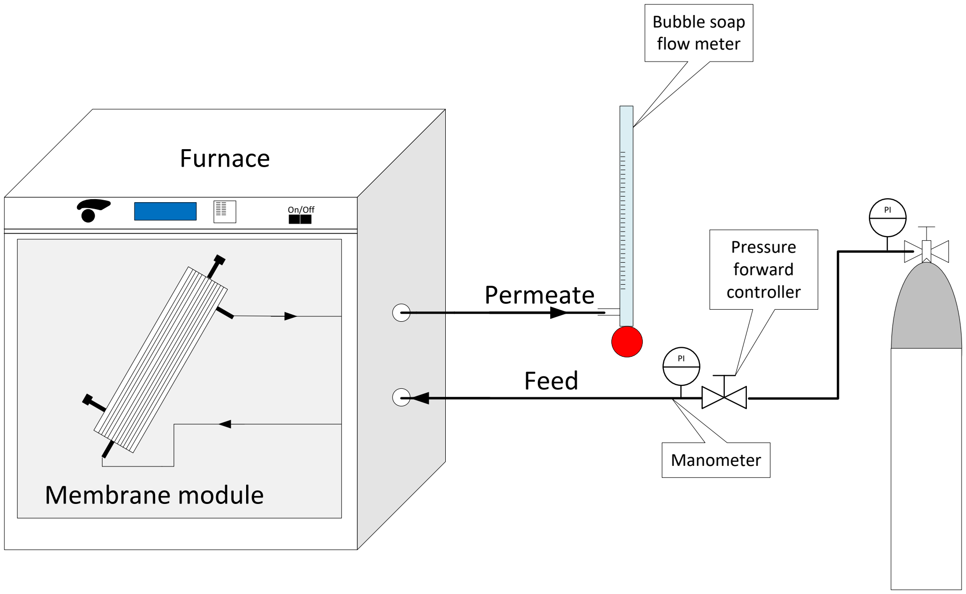

Supplementary Figure 1. Gas separation setup. A schematic showing the experimental setup for gas separation by using the pressure-drop method.

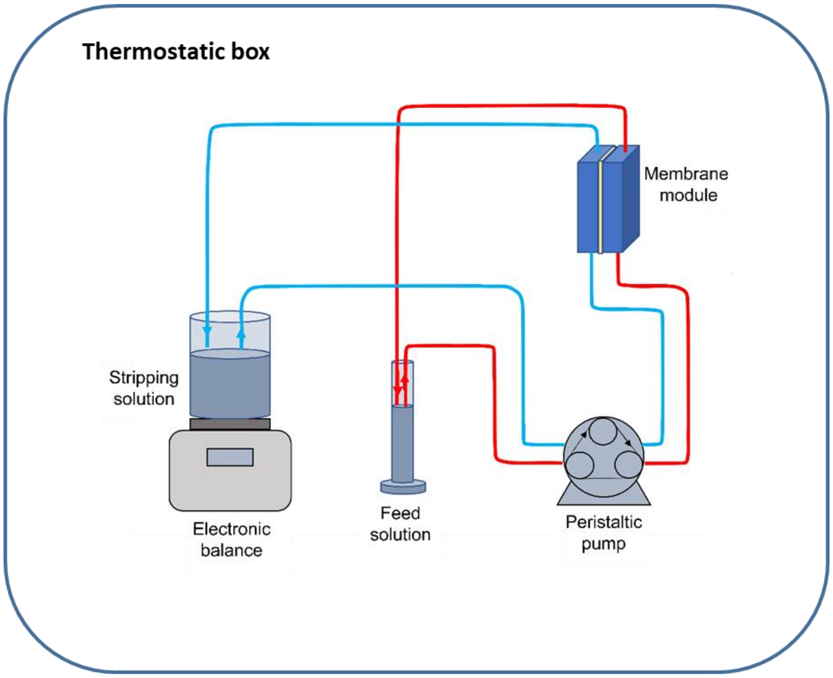

Supplementary Figure 2. Osmotic distillation (OD) setup. A schematic showing the OD system setup used to study the membrane performance.

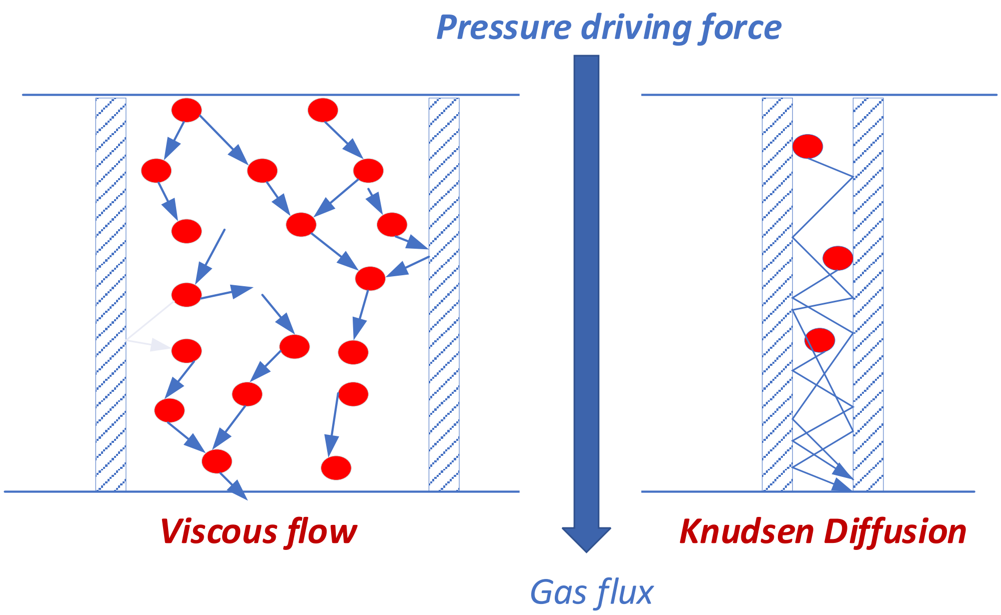

Supplementary Figure 3. Model for gas permeation. A schematic of the model for gas permeation through the membrane pores.

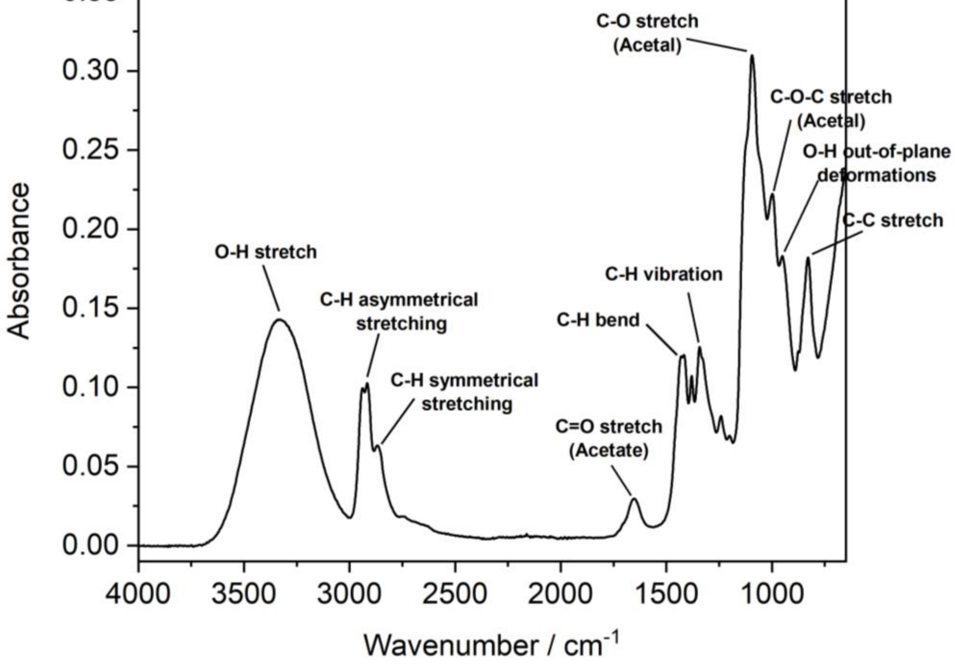

Supplementary Figure 4. Infrared spectroscopic analysis. A typical infrared spectrum, recorded in ATR mode, of a P-P membrane with an empirical assignment of the main bands.

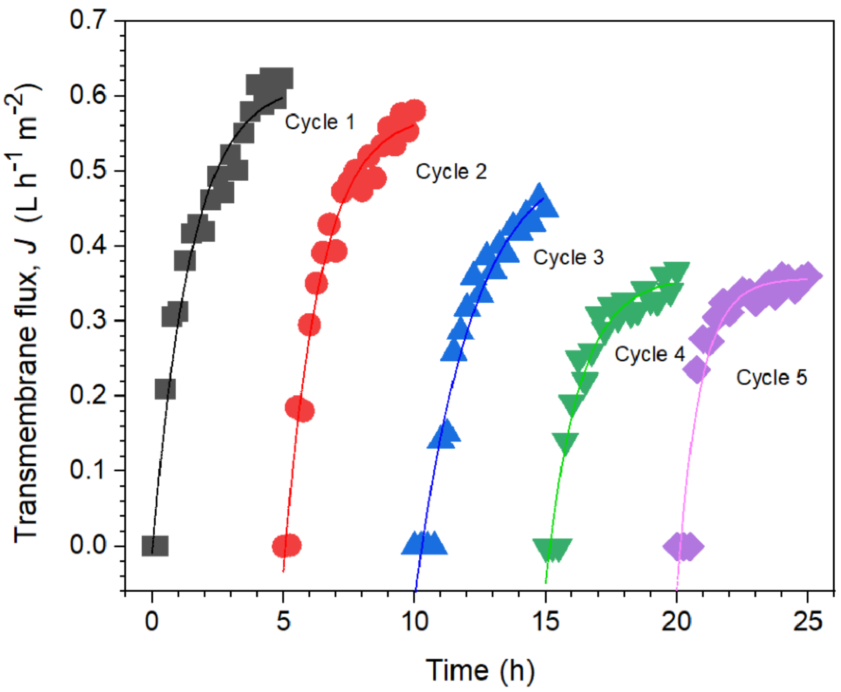

Supplementary Figure 5. Instantaneous transmembrane flux of the undoped membranes. Instantaneous transmembrane flux is shown as a function of the operating time for undoped PVDF-PVA membranes in five cycles. The solid lines are exponential fits to the experimental data points.

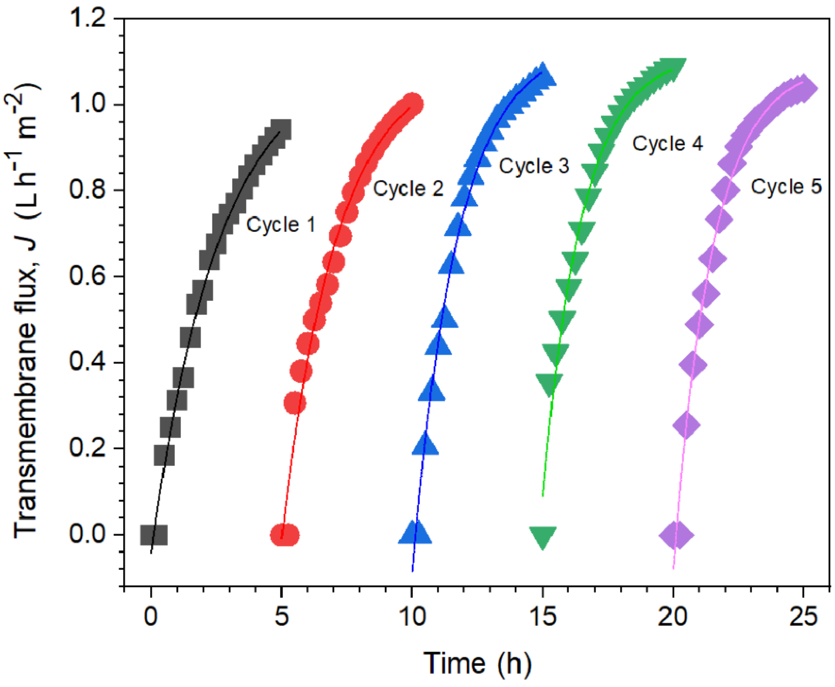

Supplementary Figure 6. Instantaneous transmembrane flux of the doped membranes. Instantaneous transmembrane flux as function of the operating time for doped PVDF-PVA-TBB membranes in five cycles. The solid lines are exponential fits to the experimental data points.

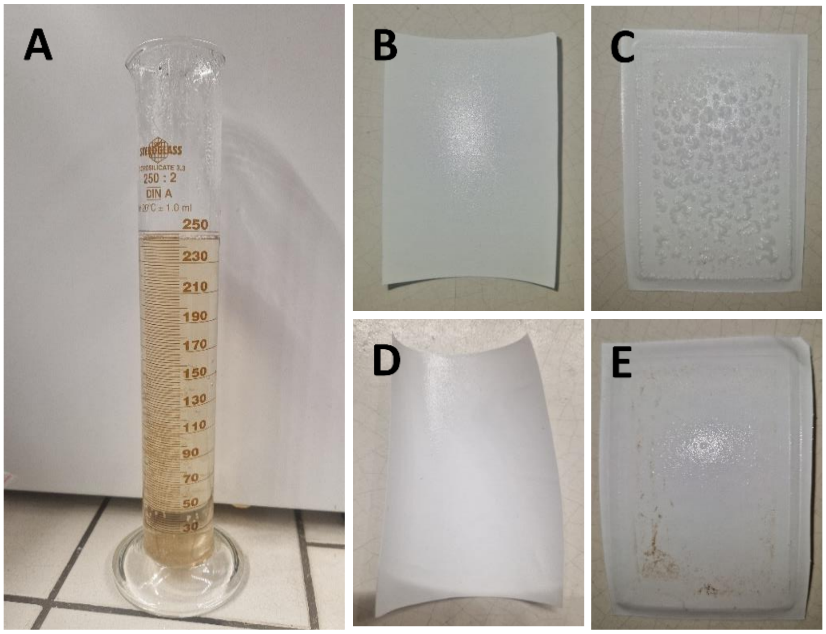

Supplementary Figure 7. Direct contact membrane distillation (DCMD) test of the membranes. (A) Hypersaline feed solution at 228 g/L TDS with composition as per Supporting Table 5. (B, C) A photograph of the P-P-T 1.0 membrane before (B) and after the third cycle (C) in the DCMD tests. (D, E) A photograph of the P-P membrane before (D) and after (E) the DCMD tests.

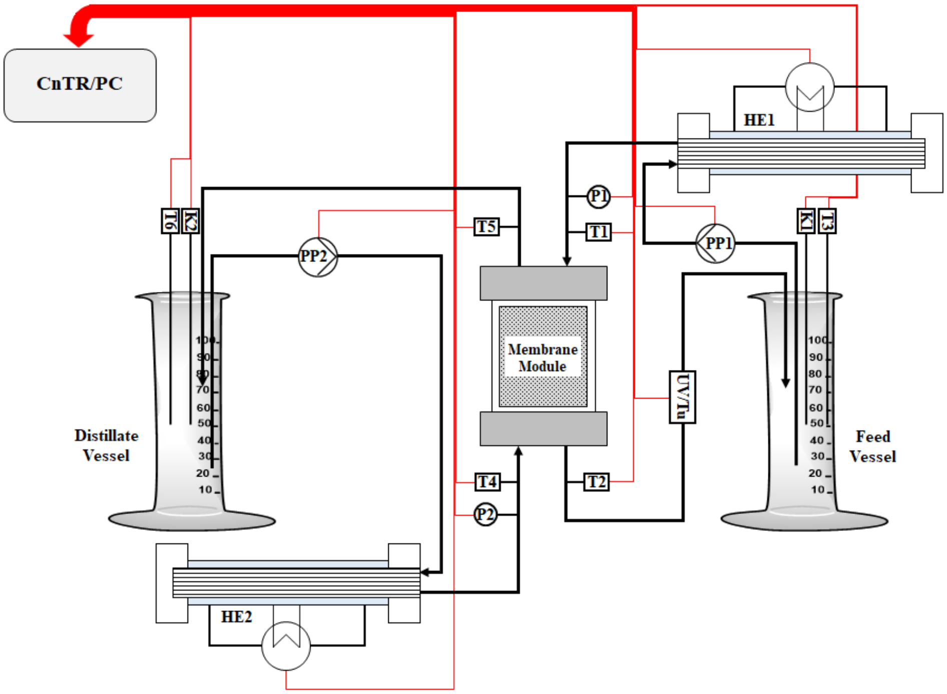

Supplementary Figure 8. Setup used for the DCMD tests. Schematic diagram of the DCMD plant composed of T1-T6 probes (PT100 from RS Components, Italy), P1-P2 pressure probes (RS Components, Italy), PP1-PP2 peristaltic pump (Masterflex L/S digital pump driver with Easy Load II pump head at two channel), HE1-HE2 heat exchangers connected to refrigerating & heating circulators (model F32, Julabo, Germany), K1-K2 conductivity meter (Jenway, Bibby Scientific, UK), membrane holder cell in nylon, UV-Tu online photometer (Pendotech SPEC-L-2-260-880-PHOTO) with 0.5 cm path length flow cell, fiber optics cables, and optical couplers (Pendotech), and a CnTR/PC logic controller (PressureMat, Pendotech) connected to a PC. The diagram was adapted with permission from reference [51]. Copyright: Elsevier, 2023.

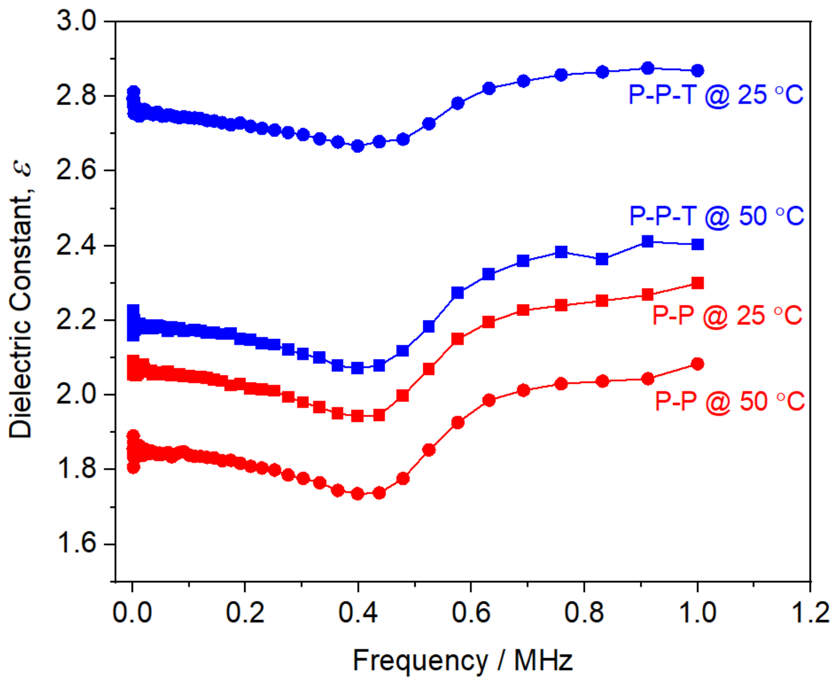

Supplementary Figure 9. Electrical characterization of the membranes. Dielectric properties measured as a function of the frequency at 25 °C and at 50 °C for pristine PVDF-PVA (P-P) membranes and doped PVDF-PVA-TBB (P-P-T) membranes.

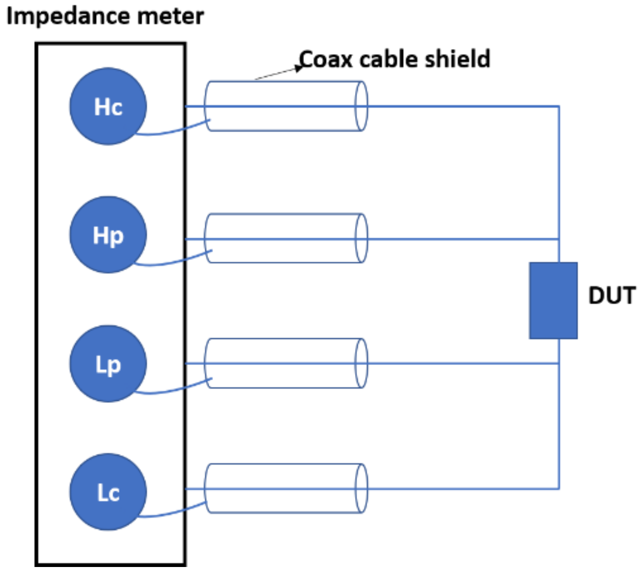

Supplementary Figure 10. Simplified configuration of the setup used for characterization of the electrical properties. A four terminal-pair measurement configuration is shown.

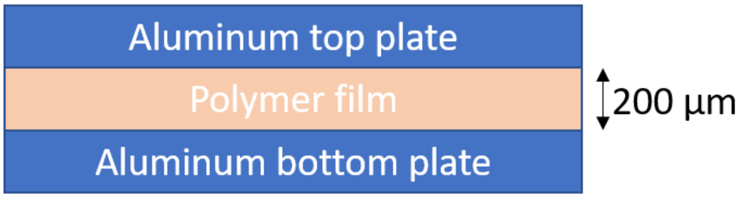

Supplementary Figure 11. Illustration of the element used to study the electrical properties. A schematic of a side view of the parallel plate capacitor used for the measurements is shown.

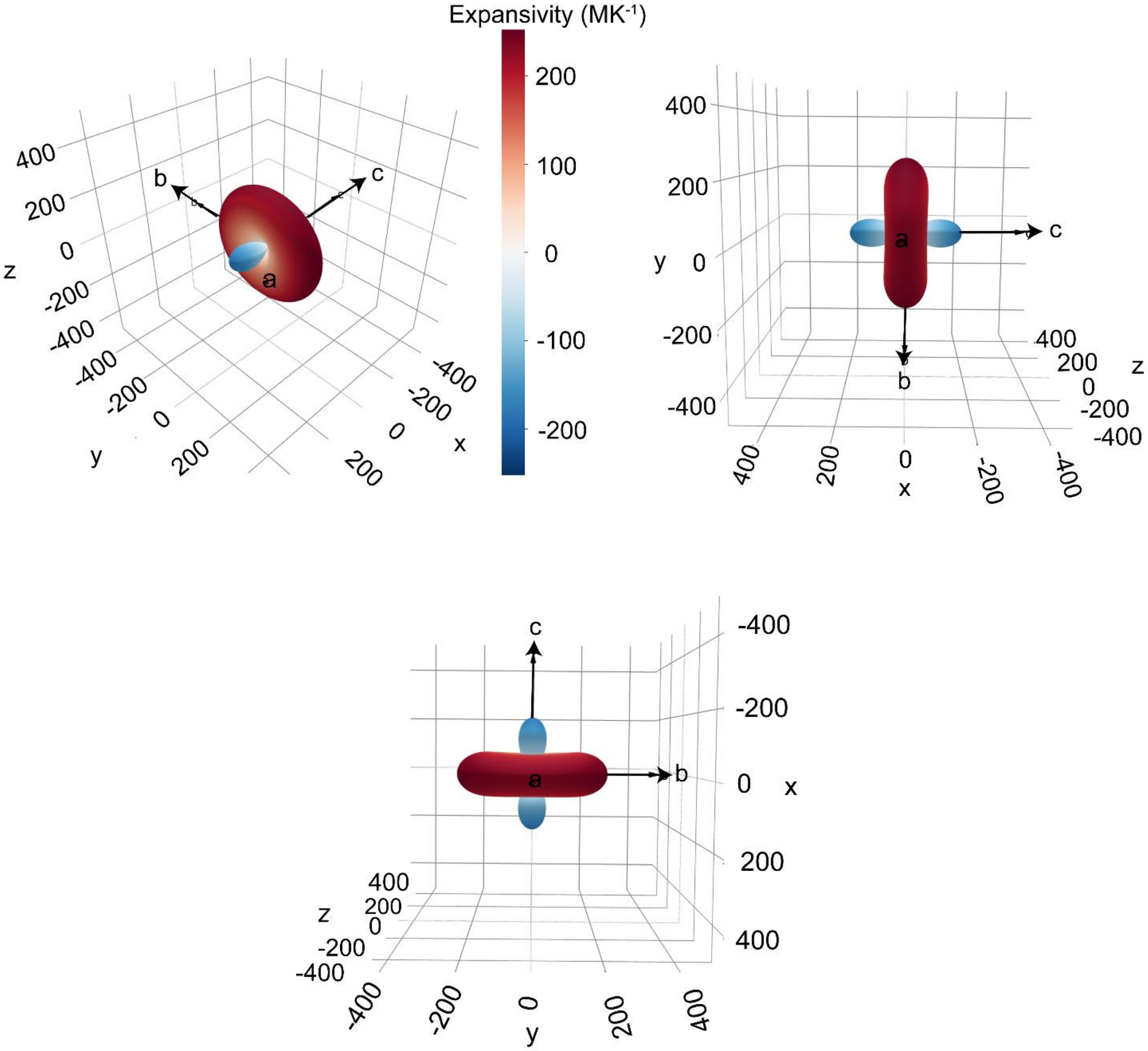

Supplementary Figure 12. Visualization of the thermal expansion of the TBB crystals. The plots show the expansivity indicatrices along the principal unit cell axes (units: MK^{-1}) for a TBB crystal.

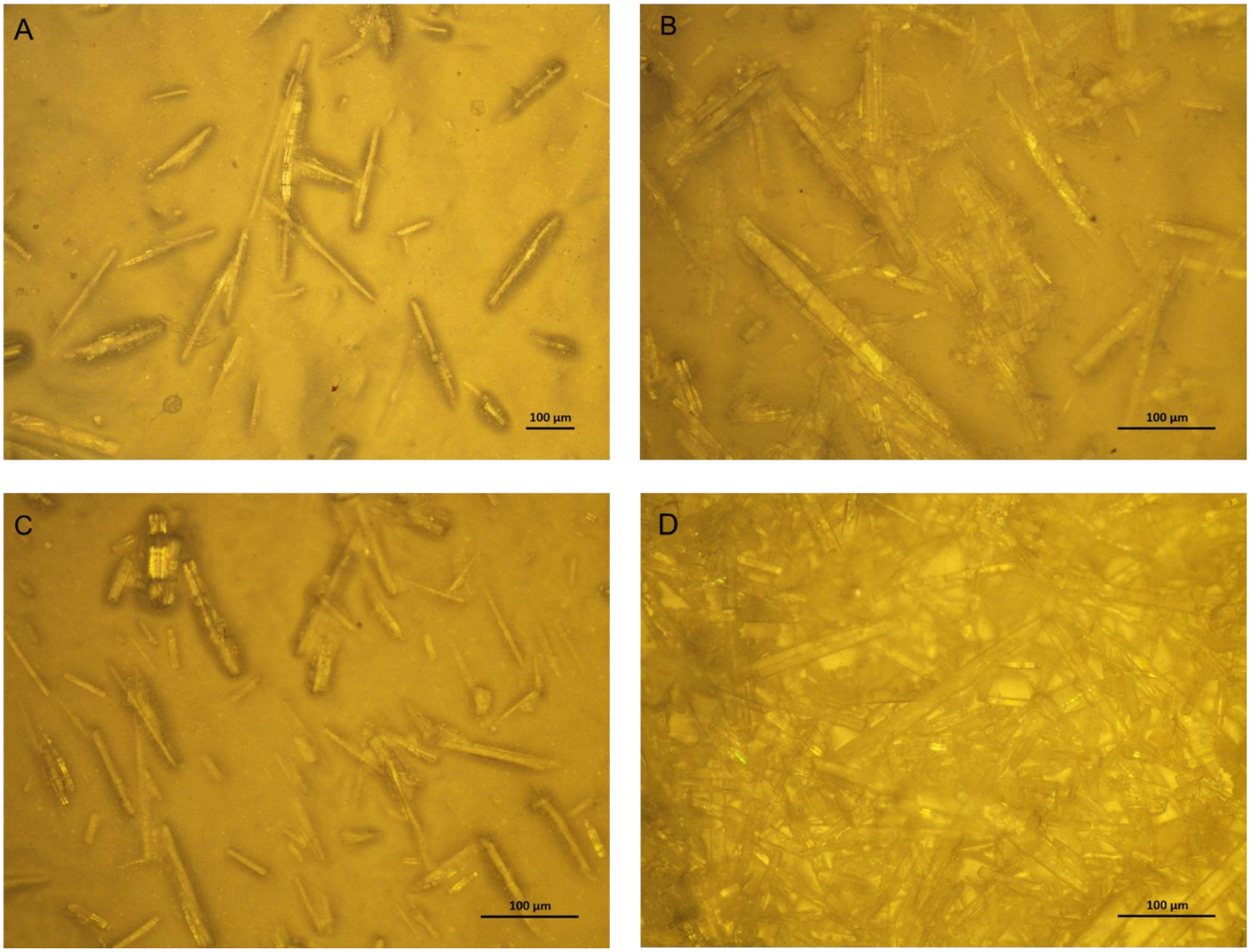

Supplementary Figure 13. Images of P-P-T membranes with different TBB loadings. A) A P-P-T membrane with a 0.1 mg cm^{-2} TBB loading, B) A P-P-T membrane with a 0.5 mg cm^{-2} TBB loading, C) A P-P-T membrane with a 1.0 mg cm^{-2} TBB loading, and D) A P-P-T membrane with a 2.0 mg cm^{-2} TBB loading.

Supplementary Tables

Supplementary Table 1. NaCl rejection (*R*%) in osmotic distillation tests with composite membranes at different TBB loadings and temperatures

Membrane	*R* (%)						
	28 °C	32 °C	36 °C	40 °C	44 °C	46 °C	48 °C
P-P	99.98	99.99	100	100	100	99.96	100
P-P-T 0.1	99.95	99.94	99.98	99.99	99.95	99.99	99.98
P-P-T 0.5	99.99	99.95	99.96	99.97	99.97	99.99	99.96
P-P-T 0.8	99.96	99.97	100	100	99.98	100	99.95
P-P-T 1.0	99.95	100	99.97	99.99	99.97	99.98	99.98
P-P-T 1.2	100	99.96	100	99.96	99.99	99.95	99.96
P-P-T 1.4	99.99	99.96	99.94	99.96	99.95	99.98	100
P-P-T 2.0	99.99	100	99.95	99.98	99.97	100	100

Supplementary Table 2. Ideal selectivity of membranes at different temperatures

Membrane	H_2/N_2 selectivity				H_2/CO_2 selectivity			
	25 °C	35 °C	40 °C	50 °C	25 °C	35 °C	40 °C	50 °C
P-P	2.78	2.72	2.67	2.63	3.05	3.25	3.26	2.79
P-P-T 0.1	2.79	2.69	2.61	2.60	2.88	2.86	2.85	2.86
P-P-T 0.5	3.31	2.82	2.75	2.65	2.31	2.23	2.19	2.12
P-P-T 1.0	3.45	2.58	2.82	2.94	2.72	2.81	2.81	3.22
P-P-T 1.2	3.92	3.14	3.26	3.22	2.84	2.90	2.28	3.31

Supplementary Table 3. Fitting parameters for transmembrane flux data points as a function of the operating time for PVDF-PVA (P-P)and PVDF-PVA-TBB (P-P-T) membranes at different reuse cycle

	PVDF-PVA				PVDF-PVA-TBB			
Cycle	A_1	t_1	y_0	R^2	A_1	t_1	y_0	R^2
1	-0.6284	-1.4757	0.6196	0.9612	-1.1576	-2.6439	1.1168	0.9953
2	-22.2909	-1.3905	0.5781	0.9748	-11.6714	-2.1264	1.1037	0.9849
3	-55.2708	-2.2109	0.5291	0.9519	-377.4134	-1.7468	1.1471	0.9918
4	-58787.4697	-1.2626	0.3591	0.9447	-21371.3584	-1.5088	1.1199	0.9863
5	-4.7007E9	-0.8639	0.3576	0.9023	-708967.9909	-1.5021	1.0969	0.9908
Fitting equation: $y = A_1 * \exp(x/t_1) + y_0$								

Supplementary Table 4. A comparison of absolute water flux and salt rejection in various types of thermoresponsive membranes

Membrane type	Absolute transmembrane flux	Rejection %	Feed	Draw agent	Feed/distillate temperature °C	Process	Note	Ref
PTFE (pore size 0.2 μm)	1.68 kg/m^2/h	99.5	NaCl 2000 ppm	P(NIPAM-MBA) hydrogel	40/NA	OMD	Thermoresponsive hydrogel	1
Cellulose triacetate (dense) with an embedded polyester screen mesh	1.81 L/m^2/h	-	NaCl 2000 mg/L	P(NIPAM-co-DEM) 5 wt% DEM hydrogel	25/NA	FO	Thermoresponsive hydrogel	2
Polypropylene (pore size 0.04 μm)	0.472 kg/m^2/h	-	apple juice 30° brix	$CaCl_2$ 6 mol/kg	29.85/NA	OD	Non-thermoresponsive process	3
Polyamide active layer (dense)/polysulfone support (porous)	1.35 L/m^2/h	99.9	NaCl 0.6 M	[P4444]TsO ionic liquid 2 M	60/NA	FO	Thermoresponsive IL	4
Cellulose triacetate (dense) with an embedded polyester screen mesh	3.1 L/m^2/h	-	NaCl 2000 ppm	PSA-1.2 wt% rGO hydrogel	45/NA	FO	Hydrogel dewatering by solar energy	5
	6.8 L/m^2/h			PSA-NIPAM-1.2 wt% rGO hydrogel	51/NA			
Polyamide (dense)	0.85 L/m^2/h	88.41	NaCl 0.17 M	ionic liquid 3.2 M	60/NA	FO	Thermoresponsive IL	6
Cellulose triacetate (dense) with an embedded polyester screen mesh	1.32 L/m^2/h	95.4	NaCl 2000 ppm	PSA-C,	25/NA	FO	Thermoresponsive hydrogel with light-absorbing particles	7
	0.69 L/m^2/h			PSA-NIPAM				
	0.77 L/m^2/h			PSA-NIPAM-C				
Cellulose triacetate (dense) with an embedded polyester screen mesh	1.08 L/m^2/h	-	NaCl 0.2 wt%	PNIPAM/γ-PGA/PEG hydrogel	40/NA	FO	Thermoresponsive hydrogel	8
Cellulose triacetate (dense) with an embedded polyester screen mesh	0.55 L/m^2/h	-	NaCl 2000 ppm	PSA-NIPAM hydrogel	25/NA	FO	Thermoresponsive hydrogel	9
Cellulose triacetate (dense) with an embedded polyester screen mesh	0.18 L/m^2/h	-	NaCl 2000 ppm	PNIPAM-IPN-PSA (SI-0.2 PSA)	25/NA	FO	Thermoresponsive hydrogel	10
	0.12 L/m^2/h			PNIPAM-IPN-PVA (SI-0.2 PVA)				
	0.18 L/m^2/h			PNIPAM-IPN-PVA (SI-0.5 PVA)				
Cellulose triacetate (dense) with an embedded polyester screen mesh	1.5 L/m^2/h	-	NaCl 2000 ppm	Sodium acrylate/N-isopropylacrylamide hydrogel with γ-Fe_2O_3	25/NA	FO	Thermoresponsive hydrogel with magnetic heating particles	11
PTFE/PP 0.25 μm	11.3 L/m^2/h	99.9	NaCl 35-100-180 g/L	NaCl,	50/20	OMD	Non-thermoresponsive process	12
	12.5 L/m^2/h			K_2CO_3				
	14.9 L/m^2/h			$MgCl_2$				
NA	NA	23	NaCl 2 g/L	NA	50/NA	Swelling/ deswellin g	Thermoresponsive PolyNIPAAm-SA hydrogel particles	13
SiO_2-PNIPAM particles on PVDF active layer/PTFE support	10.32 – 14.64 L/m^2/h	99.99	NaCl 35000 ppm	NA	60/20	MD	Thermoresponsive SiO2-PNIPAM particles for thermal membrane cleaning	14
			BSA and SA 1000 ppm in NaCl 35000 ppm					
SiO_2-PNIPAM particles on PVDF active layer/PTFE support	8 L/m^2/h	88.26	Microalgae, BSA and SA 1000 ppm in NaCl 35000 ppm	NA	60/20	MD	Thermoresponsive SiO2-PNIPAM particles for thermal membrane cleaning	15
Cellulose triacetate (dense) with an embedded polyester screen mesh	0.55 L/m^2/h	-	NaCl 2000 ppm	PEG-PLGA-PEG/GO-0.09 wt% hydrogel	30-60/NA	FO	Thermoresponsive hydrogel	16
	0.48 L/m^2/h			PEG-PLGA-PEG/GO-0.18 wt% hydrogel				
Cellulose triacetate (dense) with an embedded polyester screen mesh	0.32 L/m^2/h	-	NaCl 2000 ppm	NIPAM-SA/NIPAM bilayer hydrogel	Solar heating source (1 kW/m^2)/NA	FO	Thermoresponsive hydrogel	17
conductive poly(pyrrole) on poly(N-isopropylacrylamide)	0.045 L/m^2/h	-	0.15 g/L acidic BSA solution	NA	45	Filtration	Filtration with thermossensible deformability membranes	18
Cellulose triacetate (dense) with an embedded polyester screen mesh	2-4 L/m^2/h	-	NaCl 2000 ppm	P(NIPAM-AA) microgels	40	FO	Thermoresponsive hydrogel	19

polyamide (dense) active layer / polysulfone (porous) support	15 $L/m^2/h$	98.6	-	[N4444][TMBS] ionic liquid	60	FO	Regeneration of thermorespensive IL used in FO by high temperature RO	20
Cellulose triacetate (dense) with an embedded polyester screen mesh	4 $L/m^2/h$	-	NaCl 0.6 M	PSSS-PNIPAM hydrogel	-	FO	Integrated FO/MD with thermoresponsive hydrogel	21
PVDF (Porous)	2.7 $L/m^2/h$	-	hydrogel	NA	50/10	MD		
Polycaprolactone porous membranes with poly(N-isopropylacrylamide)	60 $L/m^2/h$	-	Oil/water emulsion	NA	50	Filtration	Thermoresponsive dead end filtration at 1-3 bar	22
Thin-film composite aquaporin membrane	2.21 $L/m^2/h$	-	NaCl 2000 ppm	NIPAMAA-SSA hydrogel	60	FO	Thermoresponsive hydrogel	23
PEBAX membrane	0.057 $kg/m^2/h$	-	nitrogen	-	30	Dehumidification		24
PEBAX/F-Ce-4%	0.082 $kg/m^2/h$							
PEBAX/IL@F-Ce-4%	0.11 $kg/m^2/h$							
PET-PNIPAAm-PA	1.0 $L/m^2/h$	33.8	NaCl 1000 ppm	NA	25	Filtration	Thermoresponsive dead end filtration at 1 bar	25
PET-PEG-PNIPAAm-PA	1.0 $L/m^2/h$	47.0		NA	25			
PET-hydrophobic PNIPAAm-PA	1.8 $L/m^2/h$	27.9		NA	45			
Wood− PNIPAM hydrogel composite	0.6-5.9 mL/min	NA	Water	NA	20-40	Filtration	Thermoresponsive dead end filtration at atmospheric pressure	26
Cellulose triacetate (dense) with an embedded polyester screen mesh	0.29 $L/m^2/h$	-	NaCl 2000 ppm	P-NIPAAm / P(NIPAAm-co-SA) hydrogel	60	FO	Thermoresponsive hydrogel	27
	0.19 $L/m^2/h$							
	0.11 $L/m^2/h$							
	0.08 $L/m^2/h$							
	0.29 $L/m^2/h$							
	0.22 $L/m^2/h$							
	0.24 $L/m^2/h$							
Cellulose triacetate (dense) with an embedded polyester screen mesh	0.26 $L/m^2/h$	-	NaCl 0.15 M	P_{4444}–DMBS ionic liquid/hydrogel	25	FO	Thermoresponsive IL and hydrogel	28
Ultrathin PEG analogue copolymer grafted SWCNTs	6340 $L/m^2/h$	23 (Au)/69 (ferritin)	Au nanoparticles and ferritin buffered in PBS	NA	40	Filtration	Thermoresponsive dead end filtration at 0.1 bar vacuum	29
PVDF-SiO_2-PNIPAm membranes	320 $L/m^2/h$	94.57	BSA 1.0 g/L solution	NA	37	Filtration	Thermoresponsive dead end ultrafiltration at 0.1 MPa	30
PES membrane with blended PNIPAM nanogels	2100 $kg/m^2/h$	NA	Water	NA	40	Filtration	Thermoresponsive dead end ultrafiltration at 0.3 MPa	31
Polycarbonate track-etched membrane with grafted PNIPAM	0.12 $mL/cm^2/s$	NA	Water	NA	40	Filtration	Thermoresponsive filtration at 0.1 MPa	32
PET polydopamine and PNIPAm modified membranes	2300 - 2500 $L/m^2/h$	80	BSA 1 mg/mL	NA	45	Filtration	Thermoresponsive filtration at 0.1-1 bar	33
PVDF/palygorskite-g-PNIPAAM	366 $L/m^2/h$	100	BSA 1.0 g/L	NA	40	Filtration	Thermoresponsive dead end ultrafiltration at 0.1 MPa	34
PES-PNIPAm-co-PGAM	10 $L/m^2/h$	20	BSA solution	NA	45	Filtration	Thermoresponsive filtration	35
PES-PNIPAm-co-PAm	15 $L/m^2/h$							
Cellulose triacetate (dense) with an embedded polyester screen mesh	0.65 $L/m^2/h$	NA	Water	Fe3O4@P(NIPAM-co-AMPS))/	25	FO	Magnetic thermoresponsive hydrogel	36
	0.11 $L/m^2/h$	NA		Fe3O4@P(NIPAM-co-AA) nanogel				
Cellulose triacetate (dense) with an embedded polyester screen mesh	45.6 $L/m^2/h$	-	NaCl 2000 ppm	N-Isopropylacrylamide-*co*-2-(diethylamino) ethyl methacrylate microgels	20	FO	Thermoresponsive hydrogel	37
Cellulose triacetate (dense) with an embedded polyester screen mesh	44.8 $L/m^2/h$	-	NaCl 2000 ppm	N-Isopropylacrylamide-co-itaconic acid microgels	25	FO	Thermoresponsive hydrogel	38
Cellulose triacetate (dense) with an embedded polyester screen mesh	2 $L/m^2/h$	-	NaCl 2000 ppm	Tributylhexylphosphonium p-styrenesulfonate P4444 SS hydrogel	25	FO	Thermoresponsive ionic liquid hydrogel	39
Cellulose triacetate (dense) with an embedded polyester screen mesh	0.30 $L/m^2/h$	-	NaCl 2000 ppm	Tributyl-4-vinylbenzylphosphonium hydrogels	20	FO	Thermoresponsive ionic liquid hydrogel	40
TFC-PSf-g-PNIPM copolymer	28.4 $L/m^2/h$	69.57	Water	NaCl 1M	25	FO	Thermoresponsive functional layer	41

poly(2-dimethylaminoethyl methacrylate)-block-poly(N-isopropylacrylamide) on PES	60 $L/m^2/h$	-	Globulin, BSA, lysozyme 1000 ppm	NA	70	Filtration	Thermoresponsive filtration at 1.2 bar	42
Thermoplastic polyurethane with poly(N-isopropylacrylamide) hydrogel	503 $L/m^2/h$	99.85	oil-in-water emulsion	NA	45	Filtration	Thermoresponsive filtration under atmospheric pressure	43
Cellulose triacetate (dense) with an embedded polyester screen mesh	0.62 $L/m^2/h$	-	NaCl 0.62 m	di(ethylene glycol) n-hexyl ether 12 m	30	FO	Thermoresponsive liquid for osmotic pressure control	44
Thin film composite membrane (dense)	1.5 $L/m^2/h$	-	NaCl 1.2 M	Tetrabutylphosphonium 2,4-dimethylbenzenesulfonate 70 wt%	32-49	FO	Thermoresponsive ionic liquid for osmotic pressure control	45
Cellulose triacetate (dense) with an embedded polyester screen mesh	1.4 $L/m^2/h$	-	NaCl 2000 ppm	NIPAM-SA hydrogel	60	FO	Thermoresponsive hydrogel	46
Cellulose triacetate (dense) with an embedded polyester screen mesh	12.1 $L/m^2/h$	-	NaCl 0.6 M	Tetraethylammonium bromide 4 M	50	FO	Thermoresponsive ionic liquid for osmotic pressure control	47
Thin film composite membrane (dense)	4.6 $L/m^2/h$	99.6	NaCl 0.6 M	Ammonium iodide salts	50	FO	Thermoresponsive ionic liquid for osmotic pressure control	48
PVDF hollow fibre membrane	2.02 $kg/m^2/h$	-	fructose/grape juice 45° brix	$CaCl_2$ 43 w/w %	55	OD	Non-thermoresponsive process	49
PP hollow fiber membrane	1.3 $kg/m^2/h$	-	kiwifruit juice 66.6° brix	$CaCl_2$ 60 w/w %	40	OD	Non-thermoresponsive process	50
PVDF-PVA-TBB	1.2 $L/m^2/h$	99.95	NaCl 0.5 M	$MgCl_2$ 35 wt%	48	OD/FO	This work	

Supplementary Table 5. Composition of the feed solution used for DCMD tests

Species	Composition [mg/L]
Cl^-	135696
Na^+	57922
K^+	20536
Mg^{2+}	9583
SO_4^{2-}	3534
Ca^{2+}	971
HCO_3^-	126
TDS	228368
BSA	100
SA	100
HA	50

Supplementary Table 6. Expansivity indicatrices along the principal axes (units: MK^{-1}) for a TBB crystal

			Direction		
Axes	α (MK^{-1})	σ_α(MK^{-1})	a	b	c
X1	-188.5373	30.6997	0.2673	-0.0	0.9636
X2	252.5461	30.2756	-0.0	1.0	-0.0
X3	234.3019	15.7555	0.9995	-0.0	0.0311
V	299.5139	22.6206			

Note: α is the linear coefficient of thermal expansion. σ_α is the error in the linear coefficient of thermal expansion. a, b and c are the projections of Xn on the unit cell axes of the TBB crystal. The unit cell parameters were taken from full data and not from the unit cell measurement alone.

Supplementary Table 7. Expansion along the principal axes of a TBB crystal. The temperature range is from 275 K to 310 K

T (K)	X_1 (%)	X_2 (%)	X_3 (%)	$X_{1,calc}$ (%)	$X_{2,calc}$ (%)	$X_{3,calc}$ (%)
275.0	-0.0	0.0	0.0	-0.04	-0.0767	0.062
280.0	-0.2199	0.1221	0.2164	-0.1343	0.0496	0.1791
285.0	-0.3143	0.0939	0.3103	-0.2285	0.1759	0.2963
290.0	-0.328	0.3096	0.3039	-0.3228	0.3021	0.4134
295.0	-0.2815	0.3096	0.7345	-0.4171	0.4284	0.5306
300.0	-0.3898	0.4032	0.6441	-0.5113	0.5547	0.6477
305.0	-0.5727	0.7488	0.7387	-0.6056	0.6809	0.7649
310.0	-0.8535	0.9351	0.8281	-0.6999	0.8072	0.882

Supplementary Table 8. Principal axes

	X1			X2			X3		
**T (K)**	**a**	**b**	**c**	**a**	**b**	**c**	**a**	**b**	**c**
280.0	-0.6367	0.0	0.7711	-0.0	1.0	-0.0	0.9833	-0.0	0.1822
285.0	-0.4681	0.0	0.8837	-0.0	1.0	-0.0	0.9895	-0.0	0.1447
290.0	0.2673	-0.0	0.9636	-0.0	1.0	-0.0	0.9995	-0.0	0.0311
295.0	0.8322	-0.0	-0.5545	-0.0	1.0	-0.0	0.9657	-0.0	0.2595
300.0	-0.4795	0.0	0.8775	-0.0	1.0	-0.0	0.9892	-0.0	0.1469
305.0	-0.2079	0.0	0.9781	0.0	1.0	-0.0	0.9948	-0.0	0.102
310.0	-0.2501	0.0	0.9682	0.0	1.0	-0.0	0.9941	-0.0	0.1084

Supplementary Table 9. Dependence of the unit cell parameters of TBB with temperature based on single crystal X-ray diffraction data

Temperature (K)	*a* (Å)	*b* (Å)	*c* (Å)	α (°)	β (°)	γ (°)
275	3.996(1)	10.644(2)	10.306(2)	90	100.73(1)	90
280	4.001(1)	10.657(1)	10.287(1)	90	100.56(1)	90
285	4.005(3)	10.654(7)	10.276(7)	90	100.54(5)	90
290	4.008(2)	10.677(5)	10.273(5)	90	100.74(4)	90
295	4.010(1)	10.677(2)	10.299(2)	90	100.21(1)	90
300	4.016(1)	10.687(3)	10.270(3)	90	100.41(2)	90
305	4.022(1)	10.724(3)	10.248(3)	90	100.48(2)	90
310	4.024(1)	10.744(2)	10.220(2)	90	100.38(1)	90

Supplementary References

1. Ren J., Li, J., Xu, Z., Du, Z. & Cheng, F. Feasibility of thermo-sensitive P(NIPAM-MBA) hydrogels as novel stripping agents for osmotic membrane distillation. *J. Environ. Chem. Eng.* **9**, 105370 (2021).
2. Bendoy, A. P. et al. Thermo-responsive hydrogel with deep eutectic mixture co-monomer as drawing agent for forward osmosis. *Desalination* **542**, 116067 (2022).
3. Ahmad, S. et al. Mass transfer modelling of hollow fiber membrane contactor for apple juice concentration using osmotic membrane distillation. *Sep. Purif. Technol*. **250**, 117209 (2020).
4. Zeweldi, H. G. et al. Forward osmosis with direct contact membrane distillation using tetrabutylphosphonium *p*-toluenesulfonate as an effective and safe thermo-recyclable osmotic agent for seawater desalination. *Chemosphere* **263**, 128070 (2021).
5. Zeng, Y. et al. Significantly enhanced water flux in forward osmosis desalination with polymer-graphene composite hydrogels as a draw agent. *RSC Adv.* **3**, 887–894 (2013).
6. Zhong, Y. et al. Using UCST ionic liquid as a draw solute in forward osmosis to treat high-salinity water. *Environ. Sci. Technol.* **50**, 1039–1045 (2016).
7. Li, D. et al. Composite polymer hydrogels as draw agents in forward osmosis and solar dewatering. *Soft Matter.* **7**, 10048–10056 (2011).
8. Zhang, K., Li, F., Wu, Y., Feng, L. & Zhang, L. Construction of ionic thermo-responsive PNIPAM/γ-PGA/PEG hydrogel as a draw agent for enhanced forward-osmosis desalination. *Desalination* **495**, 114667 (2020).
9. Li, D., Zhang, X., Yao, J., Simon, G.P. & Wang, H. Stimuli-responsive polymer hydrogels as a new class of draw agent for forward osmosis desalination. *Chem. Commun.* **47**, 1710–1712 (2011).
10. Cai, Y. et al. Towards temperature driven forward osmosis desalination using Semi-IPN hydrogels as reversible draw agents. *Water Res*. **47**, 3773–3781 (2013).
11. Razmjou, A., Barati, M. R., Simon, G. P. Suzuki, K. & Wang, H. Fast Deswelling of Nanocomposite Polymer Hydrogels via Magnetic Field-Induced Heating for Emerging FO Desalination. *Environ. Sci. Technol.* **47**, 6297–6305 (2013).
12. Zhang, et al. The role of osmotic agent in water flux enhancement during osmotic membrane distillation (OMD) for treatment of highly saline brines. *Desalination* **481**, 114353 (2020).
13. Ali, W. et al. Design of thermally responsive polymeric hydrogels for brackish water desalination: effect of architecture on swelling, deswelling, and salt rejection. *ACS Appl. Mater. Interfaces* **7**, 15696–15706 (2015).
14. Lyly, L.H.T. et al. Development of membrane distillation by dosing SiO_2-PNIPAM with thermal cleaning properties via surface energy actuation. *J. Membr. Sci.* **636**, 119193 (2021).
15. Lyly, L.H.T. et al. Desalinating microalgal-rich water via thermoresponsive membrane distillation. *J. Environ. Chem. Eng.* **9**, 105897 (2021).
16. Nakka, R. & Mungray, A.A. Biodegradable and biocompatible temperature sensitive triblock copolymer hydrogels as draw agents for forward osmosis. *Sep. Purif. Technol.* **168**, 83–92 (2016).
17. Razmjou, A., Liu, Q., Simon, G.P. & Wang, H. Bifunctional polymer hydrogel layers as forward osmosis draw agents for continuous production of fresh water using solar energy. *Environ. Sci. Technol.* **47**, 13160–13166 (2013).
18. Kim, H., Kim, K. & Lee. S. J. Nature-inspired thermo-responsive multifunctional membrane adaptively hybridized with PNIPAm and PPy. *NPG Asia Mater.* **9**, e445 (2017).
19. Hartanto, Y., Yun, S., Jin, B. & Dai. S. Functionalized thermo-responsive microgels for high performance forward osmosis desalination. *Water. Res.* **70**, 385–393 (2015).
20. Kamio, E. et al. Using reverse osmosis membrane at high temperature for water recovery and regeneration from thermo-responsive ionic liquid-based draw solution for efficient forward osmosis. *Membranes* **11**, 588 (2021).
21. Zhao, D., Wang, P., Zhao, Q., Chen, N. & Lu. X. Thermoresponsive copolymer-based draw solution for seawater desalination in a combined process of forward osmosis and membrane distillation. *Desalination* **348**, 26–32 (2014).

22. Liu, Y. et al. Thermoresponsive membranes from electrospun mats with switchable wettability for efficient oil/water separations. *Macromolecules* **51**, 8435−8442 (2018).
23. Pan, Z. et al. Sewage sludge ash-based thermo-responsive hydrogel as a novel draw agent towards high performance of water flux and recovery for forward-osmosis. *Desalination* **512**, 115147 (2021).
24. Wang, F. et al. Constructing rapid water vapor transport channels within mixed matrix membranes based on two-dimensional mesoporous nanosheets. *Commun. Chem.* **5**, 65 (2022).
25. Drikvand, H. N. et al. Thermo-responsive hydrophilic support for polyamide thin-film composite membranes with competitive nanofiltration performance. *Polymers* **14**, 3376 (2022).
26. Ding, Y., Panzarasa, G., Stucki, S., Burgert, I. & Keplinger. T. Thermoresponsive smart gating wood membranes. *ACS Sustainable Chem. Eng.* **10**, 5517–5525 (2022).
27. Zeng, J., Cui, S., Wang, Q. & Chen. R. Multi-layer temperature-responsive hydrogel for forward-osmosis desalination with high permeable flux and fast water release. *Desalination* **459**, 105–113 (2019).
28. Hsu, C. H. et al. Urban. Enhanced forward osmosis desalination with a hybrid ionic liquid/hydrogel thermoresponsive draw agent system. *ACS Omega* **4**, 4296−4303 (2019).
29. Zhu, Y., Gao, S., Hu, L. & Jin, J. Thermoresponsive ultrathin membranes with precisely tuned nanopores for high-flux separation. *ACS Appl. Mater. Interfaces* **8**, 13607−13614 (2016).
30. Zhao, Y. et al. Thermo-responsive separation membrane with smart anti-fouling and self-cleaning properties. *Chem. Eng. Res. Des.* **156**, 333–342 (2022).
31. Wang, G., Xie, R., Ju, X.-J. & Chu, L.-Y. Thermo-responsive polyethersulfone composite membranes blended with poly(n-isopropylacrylamide) nanogels. *Chem. Eng. Technol.* **35**, 2015–2022 (2012).
32. Xie, R. et al. Characterization of microstructure of poly(N-isopropylacrylamide)-grafted polycarbonate track-etched membranes prepared by plasma-graft pore-filling polymerization. *J. Membr. Sci.* **258**, 157–166 (2005).
33. Tripathi, B. P., Dubey, N. C., Simon, F. & Stamm, M. Thermo responsive ultrafiltration membranes of grafted poly(N-isopropyl acrylamide) via polydopamine. *RSC Adv.* **4**, 34073–34083 (2014).
34. Cai, J. et al. Enhanced hydrophilicity of a thermo-responsive PVDF/palygorskite-g-PNIPAAM hybrid ultrafiltration membrane via surface segregation induced by temperature. *RSC Adv.* **6**, 62186–62192 (2016).
35. Li, D., Niu, X., Yang, S., Chen, Y. & Ran, F. Thermo-responsive polysulfone membranes with good anti-fouling property modified by grafting random copolymers via surface-initiated eATRP. *Sep. Purif. Technol.* **206**, 166–176 (2018).
36. Zhou, A. et al. Magnetic thermoresponsive ionic nanogels as novel draw agents in forward osmosis. *RSC Adv.* **5**, 15359–15365 (2015).
37. Hartanto, Y. et al. Thermoresponsive cationic copolymer microgels as high performance draw agents in forward osmosis desalination. *J. Membr. Sci.* **518**, 273–281 (2016).
38. Hartanto, Y., Zargar, M., Wang, H., Jin, B. & Dai, S. Thermoresponsive acidic microgels as functional draw agents for forward osmosis desalination. *Environ. Sci. Technol.* **50**, 4221–4228 (2016).
39. Cai, Y., Wang, R., Krantz, W. B., Fane, A. G. & Hu, X. M. Exploration of using thermally responsive polyionic liquid hydrogels as draw agents in forward osmosis. *RSC Adv.* **5**, 97143–97150 (2015).
40. Fan, X. et al. Forward-osmosis desalination with poly(ionic liquid) hydrogels as smart draw agents. *Adv. Mater.* **28**, 4156–4161 (2016).
41. Salehi, H., Shakeri, A. & Lammertink, R.G.H. Thermo-responsive graft copolymer PSf-g-PNIPM: Reducing the structure parameter via morphology control of forward osmosis membrane substrates. *J. Membr. Sci.* **661**, 120794 (2022).
42. Choi, J.-Y., Yun, T. & Kwak, S.-Y. Two-step thermoresponsive membrane with tunable separation properties and improved cleaning efficiency. *J. Membr. Sci.* **554**, 117–124 (2018).
43. Ou, R., Wei, J., Jiang, L., Simon, G. P. & Wang, H. Robust thermoresponsive polymer composite membrane with switchable superhydrophilicity and superhydrophobicity for efficient oil–water separation. *Environ. Sci. Technol.* **50**, 906–914 (2016).
44. Nakayama, D. et al. Lower critical solution temperature (LCST) phase separation of glycol ethers for forward osmotic control. *Phys. Chem. Chem. Phys.* **16**, 5319–5325 (2014).

45. Cai, Y. et al. Energy-efficient desalination by forward osmosis using responsive ionic liquid draw solutes. *Environ. Sci.: Water Res. Technol.* **1**, 341–347 (2015).
46. Razmjou, A., Simon, G.P. & Wang, H. Effect of particle size on the performance of forward osmosis desalination by stimuli-responsive polymer hydrogels as a draw agent. *Chem. Eng. J.* **215–216**, 913–920 (2013).
47. Zeweldi, H. G. et al. The potential of monocationic imidazolium-, phosphonium-, and ammonium-based hydrophilic ionic liquids as draw solutes for forward osmosis. *Desalination* **444**, 94–106 (2018).
48. Park, J. et al. Systematic structure control of ammonium iodide salts as feasible UCST-type forward osmosis draw solutes for the treatment of wastewater. *J. Mater. Chem. A* **6**, 1255–1265 (2018).
49. Thanedgunbaworn, R., Jiraratananon, R. & Nguyen, M. H. Mass and heat transfer analysis in fructose concentration by osmotic distillation process using hollow fibre module. *J. Food Eng.* **78**, 126–135 (2007).
50. Cassano, A. & Drioli, E. Concentration of clarified kiwifruit juice by osmotic distillation. *J. Food Eng.* **79**, 1397–1404 (2007).
51. Fontananova, E., Grosso, V., Pantuso, E., Donato, L. & Profio, G. Di. Energy duty in direct contact membrane distillation of hypersaline brines operating at the water-energy nexus. *J. Membr. Sci.* **676**, 121585 (2023).
